# Identifying evidence-practice gaps and strategies for improvement in Aboriginal and Torres Strait Islander maternal health care

**DOI:** 10.1371/journal.pone.0192262

**Published:** 2018-02-07

**Authors:** Melanie E. Gibson-Helm, Jodie Bailie, Veronica Matthews, Alison F. Laycock, Jacqueline A. Boyle, Ross S. Bailie

**Affiliations:** 1 Monash Centre for Health Research and Implementation, School of Public Health and Preventive Medicine, Monash University, Melbourne, Victoria, Australia; 2 University Centre for Rural Health, The University of Sydney, Lismore, New South Wales, Australia; 3 Menzies School of Health Research, Charles Darwin University, Brisbane, Queensland, Australia; National Academy of Medical Sciences, NEPAL

## Abstract

**Introduction:**

Adverse pregnancy outcomes are more common among Aboriginal and Torres Strait Islander populations than non-Indigenous populations in Australia. Later in life, most of the difference in life expectancy between Aboriginal and Torres Strait Islander women and non-Indigenous women is due to non-communicable diseases (NCDs). Most Aboriginal and Torres Strait Islander women attend health services regularly during pregnancy. Providing high-quality care within these appointments has an important role to play in improving the current and future health of women and babies.

**Aim:**

This study engaged stakeholders in a theory-informed process to use aggregated continuous quality improvement (CQI) data to identify 1) priority evidence-practice gaps in Aboriginal and Torres Strait Islander maternal health care, 2) barriers and enablers to high-quality care, and 3) strategies to address identified priorities.

**Methods:**

Three phases of reporting and feedback were implemented using de-identified CQI data from 91 health services between 2007 and 2014 (4,402 client records). Stakeholders (n = 172) from a range of professions and organisations participated.

**Results:**

Stakeholders identified four priority areas relating to NCDs: smoking, alcohol, psychosocial wellbeing and nutrition. Barriers or enablers to high-quality care included workforce support, professional development, teamwork, woman-centred care, decision support, equipment and community engagement. Strategies to address the priorities included upskilling staff to provide best practice care in priority areas, advocating for availability of healthy food, housing and local referral options, partnering with communities on health promotion projects, systems to facilitate continuity of care and clear referral pathways.

**Conclusions:**

This novel use of large-scale aggregate CQI data facilitated stakeholder input on priority evidence-practice gaps in maternal health care in Australia. Evidence-practice gaps relating to NCD risk factors and social determinants of health were prioritised, and stakeholders suggested both healthcare-focussed initiatives and approaches involving the community and the wider health sector. The findings can inform health service planning, advocacy, inter-agency strategies, and future research.

## Introduction

Approximately 4% of all women giving birth in Australia are Aboriginal and/or Torres Strait Islander (12,817 in 2014) [1]. While Aboriginal and Torres Strait Islander women tend to have babies at younger ages than non-Indigenous women [1], risk factors for non-communicable diseases (NCDs) are already common among young Aboriginal and Torres Strait Islander women [2]. Smoking, high-risk alcohol use, obesity, underweight, poor nutrition and gestational diabetes are risk factors for both adverse pregnancy outcomes and NCDs that are more common among Aboriginal and Torres Strait Islander women than non-Indigenous women [1, 3–7]. This is consistent with a life expectancy of 10 years less for Aboriginal and Torres Strait Islander women, predominantly due to NCDs [5].

While many Aboriginal and Torres Strait Islander women have healthy pregnancies and babies, considerable disparities in perinatal outcomes, such as low birth weight and preterm birth, persist compared to the general population in Australia [1]. These outcomes are associated with neonatal morbidity and mortality, and may increase the risk of the baby developing NCDs in later life [8, 9]. High levels of psychological distress, and stressors during pregnancy, are common among Aboriginal and Torres Strait Islander women and may also affect the next generation through associations with low birth weight and preterm birth [10–12].

NCD risk factor prevalence in Aboriginal and Torres Strait Islander populations is associated with socio-economic disadvantage and the intergenerational effects of colonisation [5–7]. Social determinants of health and wellbeing also affect access to health care and the availability of opportunities to optimise health [6]. Aboriginal and Torres Strait Islander women are more likely than non-Indigenous women to live in remote locations and in locations of the lowest socioeconomic status [1]. However, despite these and other substantial challenges [13, 14], 86% of Aboriginal and Torres Strait Islander women attend five or more pregnancy care visits [1]. These are important opportunities to reduce disparities and improve both current and future health by providing all components of recommended maternal health care. While many aspects of maternal health care are provided at high levels by primary health centres serving Aboriginal and Torres Strait Islander communities, there is a widespread need to ensure all recommended services are delivered [15–17].

One strategy used to drive evidence-based care is Continuous Quality Improvement (CQI), a management approach that aims to constantly increase the efficiency and effectiveness of organizational systems and processes [18, 19]. Maternal health care in Australia is provided in settings that differ in characteristics such as population density, health centre governance structures, jurisdictional policies, and availability of local health and social services, and often involves a range of health professionals. For example, women in remote locations usually attend primary health care (PHC) centres for pregnancy care and then transfer to regional hospitals for the birth. Governance of Aboriginal and Torres Strait Islander specific PHC centres is either through the state/territory government health department or a community board [20].

Data collected by individual PHC centres as part of their CQI activities can be aggregated and used to identify trends across health services and over time [21], including aspects of care that are delivered at low levels by many health centres and across different settings. Such patterns are likely to represent inadequacies in the broader healthcare delivery system, and require strategies for change at multiple levels of the system rather than solely actions by individual health centres [22, 23]. Thus, the analysis of aggregated data can contribute to health service planning, policy development and future research programs if disseminated in a way that has meaning to the intended audience. However, timely and relevant translation of research evidence into policy and clinical practice is a common challenge in the health sector [24–27].

We hypothesise that one way to generate research evidence that is better matched to the needs of health services, policy makers and affected populations [25], and more likely to result in multidisciplinary strategies leading to large-scale improvement [23], is to incorporate the perspectives of multiple stakeholders when interpreting analyses of aggregated CQI data. Therefore, we aimed to facilitate stakeholder-identification of 1) priority evidence-practice gaps in Aboriginal and Torres Strait Islander maternal health care, 2) barriers and enablers to addressing such gaps and 3) strategies for achieving improvement. A secondary aim was to map the suggested strategies back to both the barriers/enablers and to the evidence-practice gaps.

## Materials and methods

### Study design

The “Engaging Stakeholders in Identifying Priority Evidence-Practice Gaps and Strategies for Improvement in PHC” (ESP) Project was designed to combine evidence on how to achieve large-scale change with knowledge co-creation [22]. This was an implementation research study design employing a theory-informed, iterative and interactive dissemination method. The theoretical basis and methods for the overall ESP Project have been reported in detail previously [22]. Feedback and experience from earlier iterations of the ESP Project (child health, chronic illness and preventive care) were used to inform the maternal health ESP process.

### Setting and context of the ESP Project: ABCD National Research Partnership

The ESP Project drew on aggregated CQI data collected as part of the Audit and Best Practice for Chronic Disease (ABCD) National Research Partnership–a collaboration that brought together PHC services, policy, support organisations and research institutions to guide and support collaborative CQI research to improve the quality of Aboriginal and Torres Strait Islander PHC across Australia. The ABCD Partnership linked more than 170 PHC centres in different parts of Australia in collaborative CQI research. The protocol and outcomes have been reported in detail previously [15–17, 21, 28] and the tools and protocols are available online [29]. Briefly, the maternal health audit tool was developed by an expert working group (e.g. medical officers, nurses and educators) and included consultation of health centre staff who would be using the tools. The audit tool was based on best practice guidelines (e.g. the Women’s Business Manual [30] and the Three Centres Consensus Guidelines on Antenatal Care [31], policy and research reports [32–36]. Between 2007 and 2014, 91 Aboriginal and Torres Strait Islander PHC centres provided clinical audit and systems assessment data on the delivery of maternal health care to the ABCD Partnership project. Participating PHC centres were located in five of Australia’s eight mainland states or territories and, reflecting the diverse settings of maternal health care in Australia, a range of locations, governance structures and population sizes were represented (S1 Table). To be eligible for inclusion in the audit, a client must: have an infant between 2 and 14 months; have been resident in the community for 6 months of the infant’s gestation; and be expected to use the health service as her usual source of PHC. The audit protocol included sampling guidelines to generate a sample likely to reflect the general population of clients [15, 28]. The evidence-based systems assessment tool guided a facilitated consensus process enabling health centre teams to evaluate the state of development of the PHC centre’s organisational systems and processes [28, 37].

### ESP Project participant recruitment: All phases

Each phase of the ESP Project used the same recruitment methods, aiming to engage stakeholders in different roles across the health care system. Stakeholders were identified by drawing on the extensive networks of the ABCD National Research Partnership, the Centre for Research Excellence in Integrated Quality Improvement and the ESP Project research team. These networks included health practitioners (e.g. doctors, midwives, Aboriginal health workers, nurses), managers and policy makers at various levels of the health system, researchers, and staff of peak bodies and support organisations that represent the interests of community-controlled health services and Aboriginal and Torres Strait Islander communities. Stakeholders were recruited by email invitation to individuals and organisations with known interest in Aboriginal and Torres Strait Islander maternal health care (e.g. community-controlled health organisations, universities and the Australian Institute of Health and Welfare), professional society newsletters (e.g. the Primary Health Care Research & Information ebulletin), and websites (e.g. Menzies School of Health Research and the Australian Indigenous HealthInfoNet). A snowballing distribution technique was used, whereby all stakeholders initially contacted were requested to forward the ESP reports and links to the online questionnaires through their networks to increase ESP Project reach. To enable engagement of those less likely to provide individual responses, we encouraged responses from facilitated group discussions.

### Ethics

Ethical approval for the ABCD National Research Partnership was obtained from research ethics committees in each relevant Australian jurisdiction [22]. All participants in the ESP Project provided individual informed consent.

### Variables, data sources and measurement

In all phases the online questionnaires could be completed by individuals or on behalf of a group of people. A group facilitation guide was provided to assist with group discussion. The first section of each questionnaire covered information about the stakeholder including their profession, organisation and jurisdiction. The specific data collection tools and methods for each phase are described in the following paragraphs.

#### Phase one: Identify and prioritise current evidence-practice gaps

In Phase One stakeholders were asked to identify priority evidence-practice gaps for maternal health care (Phase One outcome) using cross-sectional, national CQI data. The data comprised the most recent maternal health audit completed by health centres during the period 2012–2014 (n = 65 health centres; 1091 audit records; and 58 systems assessments).

Prior to stakeholder recruitment, the research team used this data to develop a preliminary set of priorities based on the following criteria:

1. Aspects of maternal health care that were generally recorded at low levels.
2. Aspects of care where there was wide variation between health centres in recorded delivery of care.
3. Aspects of care that were being delivered at high levels by most health centres, but that were being delivered at much lower levels by some health centres.
4. Organisational systems that were relatively less developed.

The data and preliminary priorities were then circulated as a report and a plain language summary with a link to an online questionnaire (S1 File). The questionnaire asked stakeholders to rate the relative importance of the 33 preliminary priority evidence-practice gaps. Stakeholders were asked to use a scale of 1–10, with 10 being the most important, however they were also able to give equal ratings to multiple priorities.

#### Phase Two: Identify barriers, enablers and strategies for addressing the prioritised evidence-practice gaps

In Phase Two stakeholders were asked to identify barriers and enablers and strategies for best practice maternal health care (Phase Two outcomes). In this phase, stakeholders were provided with longitudinal, national CQI data for the evidence-practice gaps prioritised in Phase One (from 2007 to 2014: 4402 audited records and 242 systems assessments from 91 PHC centres), and a brief on the evidence from previous quality improvement research, including research in Aboriginal and Torres Strait Islander PHC. A plain language summary of the full report, jurisdiction-specific data supplements and a link to the Phase Two questionnaire (S2 File) were also provided.

The questionnaire asked stakeholders to consider the longitudinal data, the evidence brief and their experience in PHC, and then to rate the quality of staff and health centre or system attributes relevant to supporting best practice in maternal health care, particularly relating to the priority evidence-practice gaps. A five-point Likert scale was used: strongly disagree that the attribute is in place, partly disagree, partly agree, strongly agree, I don’t know. The list of attributes that could be potential enablers (if in place) or barriers (if not in place) to improvement was drawn from international and national research [38–41] and has been published previously [38].

The questionnaire then asked stakeholders to suggest “strategies and actions that could be used to address barriers and enablers for each priority evidence-practice gap” using free-text comment boxes.

#### Phase Three: Final report preparation and feedback

A final draft report was prepared and sent to stakeholders, summarising the CQI data for the prioritised evidence-practice gaps, the barriers and enablers stakeholders agreed were important for addressing the priority evidence-practice gaps, and the strategies that stakeholders suggested. An online questionnaire was also provided, asking for any additional comments or suggestions on the presented barriers, enablers and strategies for maternal health care (Phase Three outcome) (S3 File). This provided an opportunity for stakeholders to confirm that the final report accurately reflected their responses to the Phase One and Two questionnaires.

### Data analysis

#### Analysis of questionnaire responses

Questionnaire responses were analysed after each phase so that the data could inform the next phase. Individual and group responses were analysed together. In Phase One the aspects of maternal health care that received importance ratings of 8–10 by more than 90% of stakeholders were considered the prioritised evidence-practice gaps. In Phase Two if two-thirds of stakeholders strongly or partly agreed that an attribute was in place it was considered a current enabler. If two-thirds of stakeholders strongly or partly disagreed that an attribute was in place it was considered a current barrier. For the suggested strategies (free text) one of the ESP Project team (MGH) collated and summarised the strategies and actions. A second ESP Project researcher (JB) then checked the summary against the raw questionnaire data to ensure accuracy. In Phase Three any new barriers, enablers or strategies suggested by stakeholders were incorporated into the final report by one ESP Project team member (MGH) and checked by a second (JB).

#### Synthesis of findings

One final piece of synthesis was conducted once all phases had been completed. The ESP project had guided stakeholders through a process, starting with prioritising evidence-practice gaps, then identifying barriers and enablers, and finally suggesting strategies. At each stage stakeholders were encouraged to consider the findings and their responses from the previous step so that the stages built on each other. Therefore, we hypothesised that the suggested strategies could be mapped back to both the barriers/enablers and to the evidence-practice gaps and attempted to organise the findings to map these relationships. The synthesis drew on the multidisciplinary expertise of the ESP Project team: quality improvement, Aboriginal and Torres Strait Islander health, obstetrics and gynaecology, primary health care, public health, management, knowledge translation and implementation. One of the ESP Project team members (MGH) conducted the initial mapping by hypothesising links between the suggested strategies and specific barriers/enablers and evidence-practice gaps. The ESP Project team reviewed and refined the mapping to generate the final version. This mapping allowed us to assess whether stakeholders had indeed kept the barriers and enablers in mind when discussing strategies to address the evidence-practice gaps.

## Results

### Participants

Fifty-one discrete responses, representing over one hundred stakeholders, were received across the three online questionnaires (41 individual and 10 group responses). The represented organisations included community-controlled (n = 19), government (n = 12) and general practice health services (n = 3), research organisations (n = 13), government health departments (n = 13), and health service support organisations (n = 9). Participants included nurses and midwives (n = 30), doctors (n = 18), Aboriginal and/or Torres Strait Islander Health Practitioners or Workers (n = 10), managers and board members (n = 10), CQI facilitators (n = 3), researchers (n = 10) and others (n = 8). Five individual respondents identified as Aboriginal and/or Torres Strait Islander, and all groups had some Aboriginal and/or Torres Strait Islander members. Numbers may not tally with the total number of responses, as respondents were able to select multiple answers.

### Phase One

Approximately 112 people (27 individuals and 6 group responses on behalf of 85 people) participated in Phase One.

**Fig 1:**
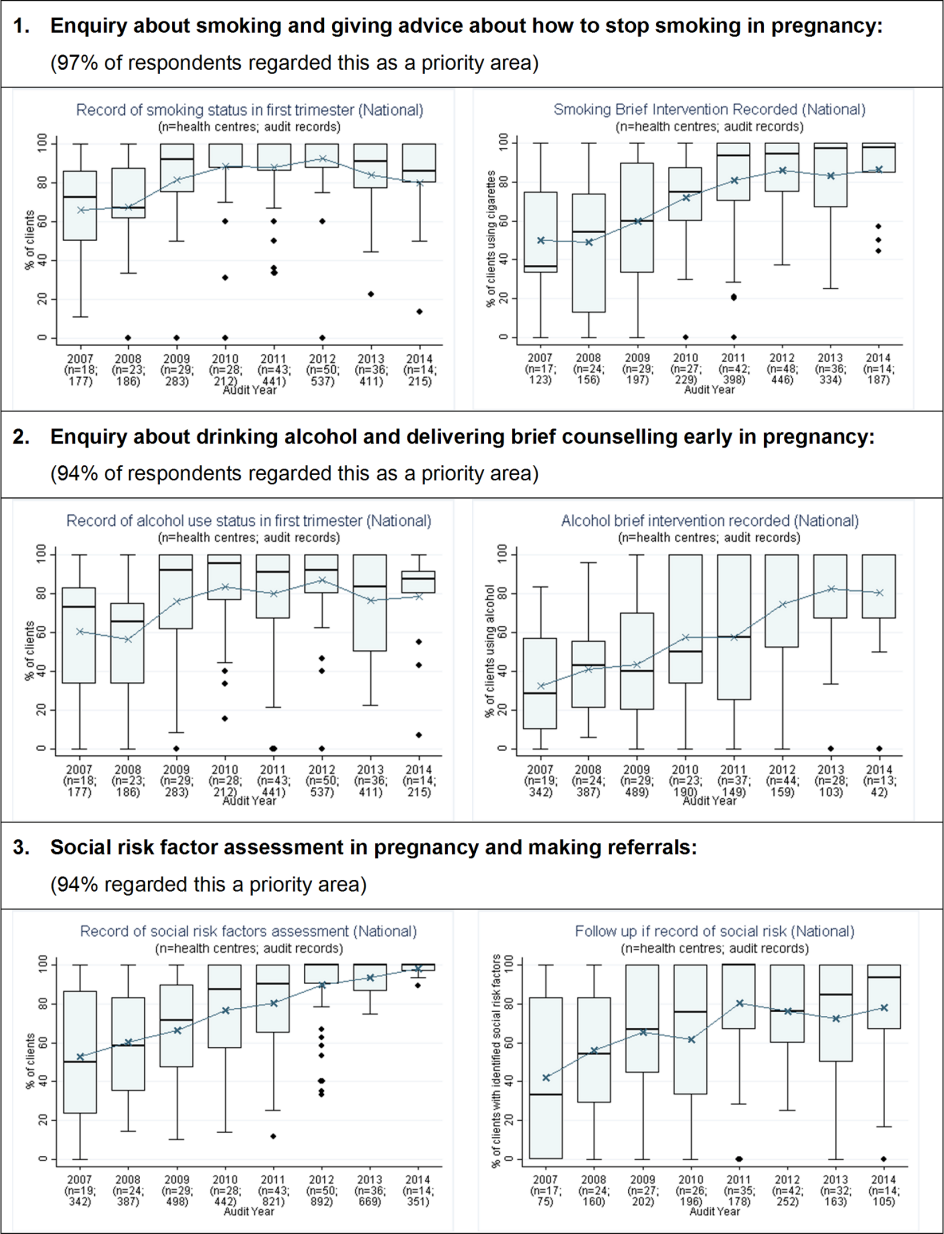
Prioritised evidence-practice gaps in Aboriginal and Torres Strait Islander maternal health care and provision of these services 2007–2014: Assessment of smoking, alcohol use and social risk factors, and subsequent brief intervention or follow-up. n = number of health centres; patient records.

**Fig 2:**
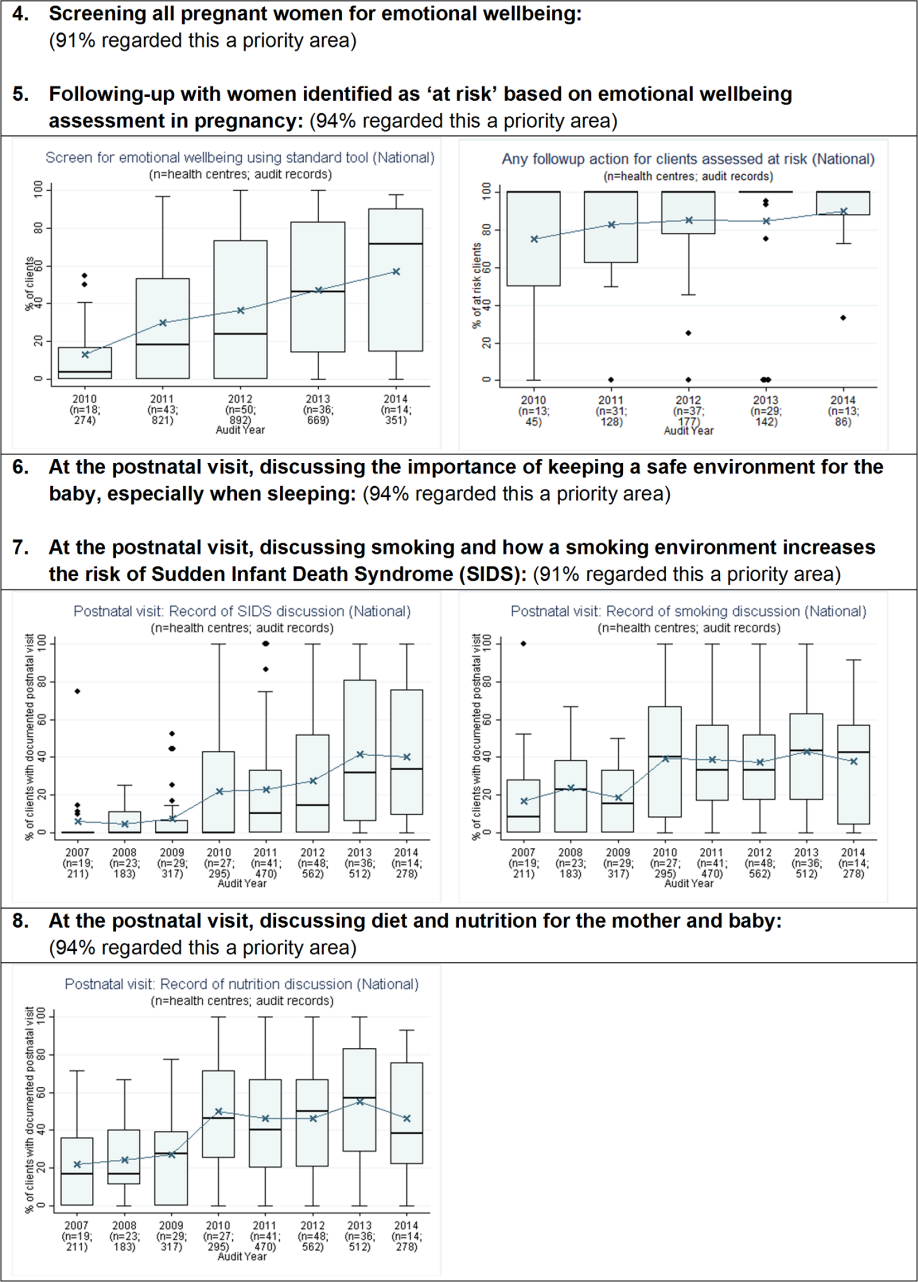
Prioritised evidence-practice gaps in Aboriginal and Torres Strait Islander maternal health care and provision of these services 2007–2014, continued: Assessment of emotional wellbeing, discussion of Sudden Unexpected Death in Infancy risk reduction, and discussion of nutrition. n = number of health centres; patient records.

The aggregated CQI data showed that many key aspects of maternal health care were provided at high levels by most health centres. Examples included laboratory investigations related to maternal immunity and infection, follow-up for abnormal glucose tests and anaemia, measurement of blood pressure, fundal height and foetal heart rate, and discussion of breastfeeding and contraception at the postnatal visit. Stakeholders identified eight evidence-practice gaps as priorities for improvement (Figs 1 and 2). These priorities related to smoking and alcohol, psychosocial wellbeing, Sudden Unexpected Death in Infancy (SUDI) and nutrition. A few stakeholders also highlighted quality of care indicators that were not captured by the audit data: family wellbeing and family support during pregnancy and at the time of birth, and preconception health care.

### Phase Two

Approximately 60 stakeholders (10 individuals and 3 group responses on behalf of approximately 50 people) participated in Phase Two. The attributes of health centres, the health system and staff that the majority of stakeholders agreed or disagreed were in place are presented in Table 1.

**Table 1:** Attributes of health centres, the health system and staff that are currently enablers or barriers to best practice maternal health care for Aboriginal and Torres Strait Islander women, particularly across the prioritised evidence-practice gaps^a^.

Health centre and health system attributes	Agreed already in place	Attribute domain
**Current enablers (attributes that are already in place)**		
Systems to support staff development.	9/13 respondents^b^	Training and development
PHC staff function effectively in teams.	9/13	Teamwork
PHC staff are clear about their roles in relation to other team members.	9/13	
PHC centres generally have appropriate numbers of:		
• medical specialists	9/13	Staffing/workforce support, recruitment and retention
• administrative staff	10/13	
Systems to recruit, retain and support general practitioners.	9/13	
PHC centres generally have adequate equipment.	8/12	Equipment, finance, resources and facilities
Availability of best practice guidelines and other decision support resources.	8/11	Decision support
Staff are adequately trained to use these resources.	8/11	
**Current barriers (attributes that require strengthening)**		
Systems to:		Staffing/workforce support, recruitment and retention
• Ensure appropriate numbers of Aboriginal and/or Torres Strait Islander Health Practitioners /Workers and midwives.	2/13	
• Recruit, retain and support Aboriginal and/or Torres Strait Islander Health Practitioners /Workers.	3/13	
• Ensure staff have support from experienced staff, especially when health centres are affected by staff turnover and shortages.	2/12	
Systems that support all PHC staff to:		Patient-centred care
• Understand the needs and aspirations of people living in Aboriginal and Torres Strait Islander communities for the purpose of providing best practice maternal health care.	3/11	
• Provide care that is respectful of and responsive to individual patient preferences, needs and values and for patient values to guide all clinical decisions.	2/11	
Systems to:		Community capacity, engagement and mobilisation
• Increase the expectation of community members regarding best practice care.	2/10	
• Enhance the health literacy of community members.	1/10	
• Build the capability of PHC staff and to support them to develop effective links and to work in partnership with communities.	2/10	
**Current staff attributes**	**Agreed already in place**	**Attribute domain**
**Enablers**		
Staff are confident in their ability to provide best practice care.	8/9	Beliefs about capabilities
Staff know the content and objective of best practice care.	7/9	
Staff know how to provide best practice care.	6/9	Knowledge
Staff recognise the professional responsibility to provide best practice maternal health care for Aboriginal and Torres Strait Islander women.	7/9	Professional identity
Staff believe that best practice maternal health care will have benefits at a population level.	7/9	Beliefs about consequences
Staff often or always remember to provide best practice care.	7/9	Memory, attention and decision processes
Staff have no trouble focussing their attention on providing best practice care.	7/9	
Staff believe that most people of influence in health centres are seen to support provision of best practice maternal health care to Aboriginal and Torres Strait Islander women.	7/9	Social influences
Staff have the skills to provide best practice care.	6/9	Skills
Staff are optimistic about the future with regard to providing best practice maternal health care for Aboriginal and Torres Strait Islander women.	6/9	Optimism
Staff have a very strong intention to provide best practice care every day.	6/9	Intentions

^a^Smoking and alcohol, psychosocial wellbeing, Sudden Unexpected Death in Infancy and nutrition.

^b^Actual number of stakeholder views reflected here is higher (up to approximately 60 people) than the presented denominator as each group response is only included once.

Enablers of best practice maternal health care, particularly regarding the priority evidence-practice gaps, were systems that support adequate health centre equipment, staff training and development, teamwork, decision support resources and systems for health centre staffing and workforce support for some staff types (Table 1). Systems requiring strengthening in order to better support best practice maternal health care included systems to build and support the Aboriginal and/or Torres Strait Islander workforce, woman-centred care, and community capacity and engagement (Table 1). The attributes of staff in place to support best practice maternal health care, as relevant to the priority evidence-practice gaps, reflected the domains of knowledge, skills, professional identity, beliefs about capabilities, optimism, beliefs about consequences, intentions, memory, attention and decision support, and social influence (Table 1).

**Fig 3:**
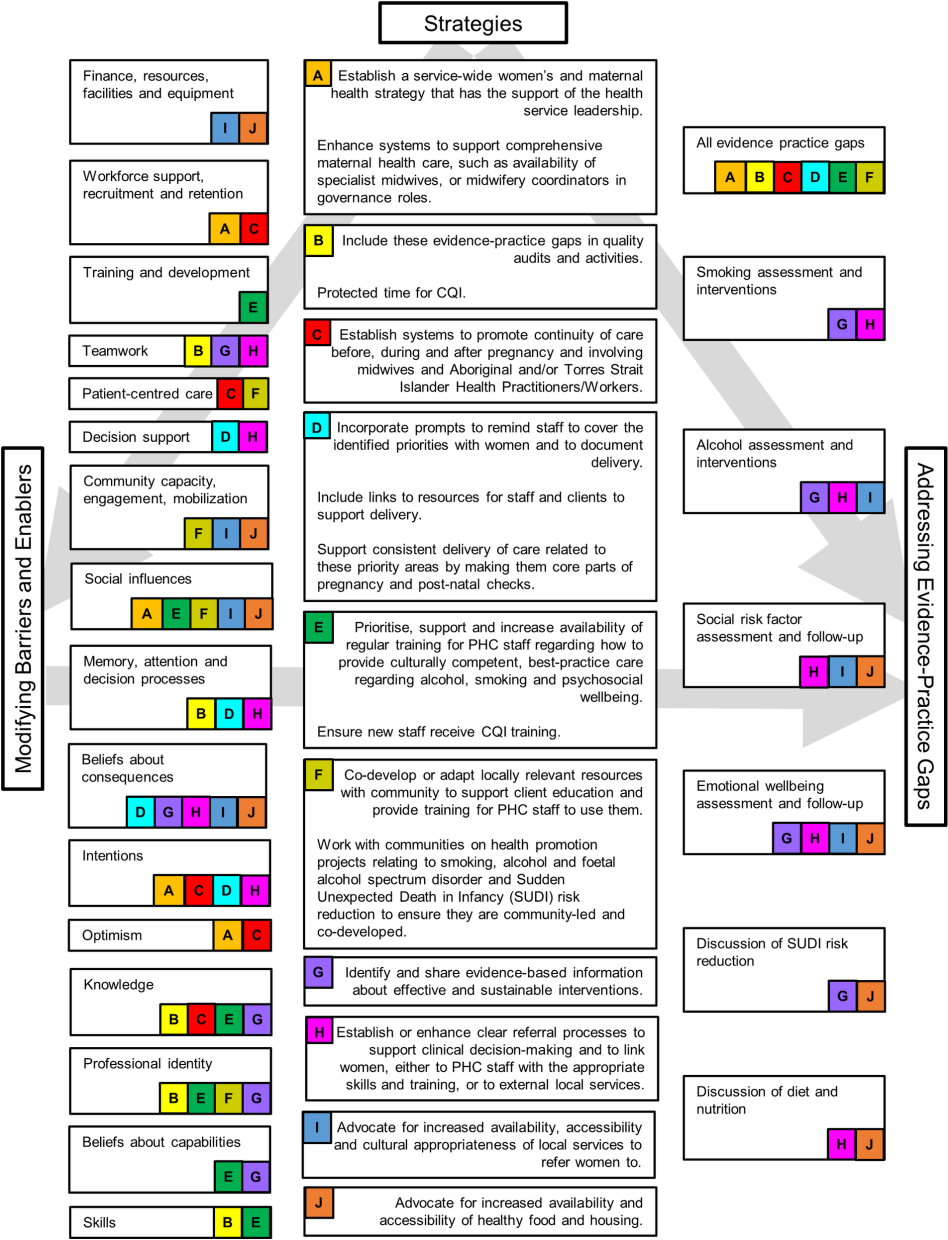
Strategies to address the barriers and enablers for each prioritised evidence-practice gap. Centre column: Strategies or actions suggested by stakeholders during Phase Two (labelled A-J). Left column: attributes of health centres, the health system and staff identified as barriers or enablers during Phase Two. Letters A-J indicate which strategies map to these attributes. Right column: Evidence-practice gaps prioritised during Phase One. Letters A-J indicate which strategies map to these priority areas.

Stakeholders used the free-text sections to provide context for their ratings of health centre, system and staff attributes, and to suggest strategies to address barriers and enablers for each prioritised evidence-practice gap. The suggested strategies are summarised in Fig 3 (centre column, labelled A-J). Some stakeholders specifically noted the importance of understanding the social determinants of health of each woman and in her local area: housing availability and conditions, food availability and accessibility, transport, and safety at home. A related theme was the availability of effective, practical and sustainable risk factor interventions. The importance of midwives in conducting and coordinating maternal health care for both well women and women with complex care needs was also emphasised. A need specifically noted was for adequate systems to support the wellbeing of the workforce, particularly around workloads, time, backfill and reflective practice.

Some strategies addressed health centre and staff attributes such as training, continuity of care, and processes to support consistent care. Other strategies built on the existing strong staff attributes but require partnerships across the broader health system such as enhancing referral processes, advocating for local services and working with communities on health promotion and education projects. An overarching theme across all strategies was the importance of working with communities and in a culturally appropriate manner.

### Phase Three

Five responses were received regarding review of the draft final report (4 individuals and 1 group response on behalf of 5 people), providing feedback from nine people in a variety of roles. An additional strategy suggested at this stage was to take a long-term funding approach to midwifery-led maternal health care and Aboriginal and Torres Strait Islander women’s health care to enable successful temporary services or programs to continue. A second suggested strategy was to strengthen systems to recruit, retain and support doctors and nurses, especially Aboriginal or Torres Strait Islander doctors and nurses, with language skills and cultural knowledge. All respondents indicated that the ESP process had improved their understanding of how to use aggregated CQI data to inform decision-making.

### Synthesis of findings

As hypothesised, the strategies (centre column of Fig 3) could be mapped to attributes of health centres, the health system and individuals that stakeholders had identified as current barriers or enablers (left column of Fig 3) and also mapped to the evidence-practice gaps (right column of Fig 3). All of the strategies could be mapped to more than one priority area and to both individual and higher level attributes. For example, advocating for local services to refer women to (strategy I) could build on the belief that best practice care will have benefits at the population level (beliefs about consequences) and could also increase the expectation of community members regarding best practice care (community capacity, engagement and mobilization). Ultimately, this strategy could address several prioritised evidence-practice gaps by improving provision of follow-up for multiple social determinants of health (high-risk alcohol use, social and emotional wellbeing).

## Discussion

### Summary of findings

This research engaged a range of stakeholders across the health sector to identify eight priority evidence-practice gaps in Aboriginal and Torres Strait Islander maternal health care. These gaps related to smoking, alcohol, nutrition, and social and emotional wellbeing. Current enablers of best practice care included decision support resources and systems that support adequate equipment, training and development, and teamwork. Many staff attributes were also identified as current enablers. Systems perceived to be less developed (and therefore current barriers) included those that support the Aboriginal and/or Torres Strait Islander workforce, woman-centred care, and community engagement. Strategies to address the evidence-practice gaps related to training, referral processes, continuity of care, processes to support consistent and comprehensive care, advocacy for availability of local services and working with communities on health promotion and education projects.

### Interpretation and comparison with existing literature

Aboriginal and Torres Strait Islander women are populations at increased risk of NCDs and many young women will enter pregnancy with existing NCDs or NCD risk factors. Six of the eight prioritised evidence-practice gaps directly related to NCD risk factors and social determinants of health. Therefore, we conclude that stakeholders in this research recognised these elevated risks and the important role of maternal health care in reducing NCD risk for both mothers and babies. Addressing social determinants of health requires both improving health care at individual health centres and broader approaches. Therefore, it’s not surprising that some strategies addressed attributes of individuals and health centres or the health system. However, such strategies require partnerships with communities, external services and policy makers. This fits with the hypothesis that evidence-practice gaps across many health centres are due to failures of the wider health system [22] and require a combined program of strategies simultaneously targeting different parts of the health system [23]. Recent diabetes care research identified different patterns of delivery and improvement for different types of care processes (e.g. laboratory tests, patient education) [42], supporting the need for action at different levels of the heath system.

As shown in Fig 3, stakeholders often identified the same or similar strategies to address different evidence-practice gaps, such as training and upskilling staff to have conversations with women about smoking, alcohol use and emotional wellbeing (strategy E). This is consistent with the focus of CQI on the functioning of organisational systems and identifying root causes rather than on isolated problems, individual components of care or individual staff [18, 19]. The barriers and enablers identified here, such as systems and processes that support woman centred-care, staff training and development, and multidisciplinary teamwork, are consistent with a review identifying key organisational elements that facilitate high-quality performance in PHC [43]. One notable difference is the additional emphasis that stakeholders in our research placed on strengthening systems that support community capacity, engagement and mobilisation. This may reflect the setting of the ESP Project (PHC for Aboriginal and Torres Strait Islander populations) but is likely to be relevant to PHC for other culturally and linguistically diverse populations. Some of the barriers and enablers identified in our research were the same as those identified in other areas of Aboriginal and Torres Strait Islander PHC [38], yet there were also some differences, perhaps reflecting the views of a different sample of stakeholders or the qualitative differences between maternal health care and care for disease states.

There is a growing emphasis on the importance of meaningful stakeholder involvement in setting research priorities and co-creation of implementation research projects, both generally and specifically in PHC [44–48]. The process described here adds to this developing field by using large-scale data regarding health care delivery as a starting point for priority setting. The finding that each strategy could be matched to barriers/enablers and to evidence-practice gaps indicates that stakeholders drew on both when proposing strategies. This suggests the process was consistent with the implementation research approach of using evidence (the CQI data) and theory (individual and higher level attributes) to develop strategies for change [49]. However, further formal evaluation of the ESP program is needed to confirm this. Best practice in research priority setting and healthcare improvement also includes consultation of patients [44]. This aligns with our finding of a need to strengthen systems that support community engagement.

### Implications for future research and clinical practice

Dissemination of research evidence and timely implementation in policy and clinical practice is a key challenge for researchers, policy makers, health services and health professionals [24–26]. Our findings can be used for health service planning. By matching service performance data to common barriers and enablers and then to possible strategies, the new insights generated here can contribute to operational planning at health centres. At a strategic planning level, the findings are a call to action for health services to commit to a maternal health strategy that recognises the critical role of comprehensive maternal health care in improving pregnancy outcomes in the short term and reducing NCD risk for both mothers and babies in the long term.

The findings can also be a starting point for strategies to be developed collaboratively across different levels of the health system. For many of the prioritised gaps a key strategy was to improve the availability of local, appropriate services and resources to support health services to take a life-course and woman-centred approach to maternal health care. The findings relating to current service performance data and stakeholder-identified barriers and enablers address a challenge to evidence uptake frequently reported by policy makers: inadequate access to timely, relevant and high-quality research evidence [27].

The ESP Project process created an important opportunity for stakeholders to contribute their perspectives to research. Capturing these perspectives enables researchers to focus on areas important to people involved in health service delivery. The process and findings may facilitate researchers and stakeholders working together to develop research programs with real world application.

### Strengths and limitations

As barriers exist across multiple levels of the health system, we included questions on broader health centre and system attributes in the Phase Two questionnaire. These additional questions have not been validated like the questions relating to individual attributes. Another limitation is that recruitment relied on stakeholders passing on the invitation through their networks, so it is not possible to accurately measure reach or response rates. Caution is needed when interpreting the findings from the Phase Two questionnaire as relatively fewer responses were received, which limits generalisability.

One of the main strengths of this research is that it used the largest and most comprehensive longitudinal dataset relating to Aboriginal and Torres Strait Islander maternal health care provision. However, it is important to note that the dataset captured recorded delivery and may underestimate actual service delivery due to under-documentation. Another strength is that a range of stakeholders was recruited and so the findings reflect knowledge of front-line health workers and others working within the health system.

## Conclusion

This novel study used large-scale aggregated CQI data and a theory-informed dissemination strategy to engage stakeholders involved in Aboriginal and Torres Strait Islander maternal health care in Australia. Stakeholders identified priority evidence-practice gaps in maternal health care relating to NCD risk factors and social determinants of health, some barriers and enablers associated with these gaps, and strategies for achieving improvement. These findings can be used in health service planning, advocacy efforts, development of inter-agency strategies, and to inform the maternal health care research agenda.

## Supporting information

S1 File Phase One survey to identify and prioritise current evidence-practice gaps in Aboriginal and Torres Strait Islander maternal health care. (PDF)

S2 File Phase Two survey to identify barriers, enablers and strategies for addressing the prioritised evidence-practice gaps. (PDF)

S3 File Phase Three survey asking stakeholders for confirmation that the final report accurately reflected their responses to the previous surveys. (PDF)

S1 Table Characteristics of PHC centres providing maternal health audit data from 2007 to 2014. PHC: primary health care, CQI: continuous quality improvement, n: number.
(DOCX)
