# Supplementary material for: Identifying evidence-practice gaps and strategies for improvement in Aboriginal and Torres Strait Islander maternal health care
Source: PLoS One. 2018 Feb 7;13(2):e0192262. doi: 10.1371/journal.pone.0192262 (PMC5802899; doi:10.1371/journal.pone.0192262)
Supplement: S1 File — (PDF) [file pone.0192262.s001.pdf]

Introduction

The survey has been developed by the ABCD National Research Partnership to identify priority evidence-practice gaps in maternal health care in Australia.

This is the first phase in a process to encourage participation in the analysis and interpretation of aggregated CQI data on maternal health care. The next phase will focus on barriers and enablers to improving care, and improvement strategies.

Prior to completing this survey you should be familiar with the information contained in the Phase 1 report: *'National Report on Aboriginal and Torres Strait Islander Maternal Health Care 2012 – 2014'.* We have also developed supplementary data tables for those jurisdictions where there are sufficient data available to allow for comparison with the national report.

The ABCD National Research Partnership is being conducted with the approval of research ethics committees in each of the jurisdictions where there are participating health centres.

This survey will take about 20 minutes to complete.

* 1. By clicking 'Yes' here, you consent to take part in this survey. Your responses will remain confidential.

- ○ Yes
- ○ No

2. If you would like to receive feedback about this research and have the opportunity to take part in further phases, please provide your contact details:

Name: ____________________

Email address: ____________________

If you wish to supply your details but not have them linked to your survey response, contact us separately at abcd@menzies.edu.au

NATIONAL RESEARCH PARTNERSHIP | Improving practice through research
Maternal Health Phase 1

Background Information

Please provide some information about yourself.

* 3. For which jurisdiction are you providing feedback? (select all that apply)

- [ ] All of Australia
- [ ] ACT
- [ ] New South Wales
- [ ] Northern Territory
- [ ] Queensland
- [ ] South Australia
- [ ] Tasmania
- [ ] Western Australia
- [ ] Victoria

* 4. What is the location of the population group you are considering in your feedback? (select all that apply)

- [ ] Urban
- [ ] Regional
- [ ] Remote

* 5. Are you responding on your own behalf, or on behalf of a group?

- ○ On my own behalf
- ○ Group

ABCD NATIONAL RESEARCH PARTNERSHIP | Improving practice through research

Maternal Health Phase 1

Group Information

* 6. **If you are responding on behalf of a group**
How many people (approximately) are in the group?

* 7. What proportion of the group identify as Aboriginal and/or Torres Strait Islander?

- All
- More than half
- Less than half
- None

* 8. Select roles represented by group members (select all that apply)

- Nurse
- Midwife
- Middle manager
- Medical Officer/Doctor
- Public health physician
- Other medical specialist
- Senior manager/Executive
- CQI facilitator
- Board member
- Policy officer
- Aboriginal and/or Torres Strait Islander health practitioner/worker
- Researcher/Academic
- Other

Other (please specify)

* 9. What types of organisations do the members of the group represent? (select all that apply)

- [ ] Community controlled health centre
- [ ] Community controlled peak body
- [ ] Government health centre
- [ ] Government health department
- [ ] Primary Health Care Network
- [ ] General Practice
- [ ] University or Research Organisation
- [ ] Other

Other (please specify)

ABCD NATIONAL RESEARCH PARTNERSHIP | Improving practice through research

Maternal Health Phase 1

Individual Information

* 10. If you are responding as an individual:

Do you identify as being of Aboriginal and/or Torres Strait Islander origin?

- ( ) Yes
- ( ) No

* 11. What is your primary role?

- ◯ Nurse
- ◯ Midwife
- ◯ Middle manager
- ◯ Medical Officer/Doctor
- ◯ Public health physician
- ◯ Other medical specialist
- ◯ Senior manager/Executive
- ◯ CQI facilitator
- ◯ Board member
- ◯ Policy officer
- ◯ Aboriginal and/or Torres Strait Islander health practitioner/worker
- ◯ Researcher/Academic
- ◯ Other

Other (please specify)

* 12. What types of organisations do you represent?

- ☐ Community controlled health centre
- ☐ Community controlled peak body
- ☐ Government health centre
- ☐ Government health department
- ☐ Primary Health Care Network
- ☐ General Practice
- ☐ University or Research Organisation
- ☐ Other

Other (please specify)

The next set of questions asks you to rate the relative importance of the preliminary priority evidence-practice gaps (or priorities for improvement) we have identified from the data. These are detailed in the Phase 1 report: 'National Report on Aboriginal and Torres Strait Islander Maternal Health Care 2012 – 2014' , and are sumarised below:

1. **client records and early pregnancy care,** including folate prescription prior to conception, pregnancy care visits before 13 weeks and completeness of delivery summaries
2. **risk factors and brief interventions,** such as documented discussion of smoking and alcohol use with all women, and brief intervention, counselling or referral for social aspects of maternal wellbeing
3. **laboratory investigations and follow-up**, particularly offering foetal anomaly screening to all women and consistent provision of anti-D injections and rubella immunisations for women who require them
4. **routine antenatal checks and follow-up of abnormal findings** , particularly calculating body mass index and providing body mass index management plans for all women, follow-up mid-stream urine (MSU) tests after abnormal urinalysis tests, and discussing foetal movements and performing a cardiotocograph (CTG) or initiating kick charts for women who report decreased foetal movements
5. **emotional wellbeing screening** for all women and appropriate follow-up for women identified to be at-risk
6. **postnatal care** including discussing sudden infant death syndrome (SIDS) prevention, and social circumstances such as finance, housing and food security at postnatal visits
7. **health centre systems**, particularly structures and processes that support continuity of care, specialist-generalist collaborations, education, behavioural risk reduction and self-management support. Communication and cooperation on health centre governance and operation, and on regional planning and development of health resources were also identified as specific priorities for improvement.

ABCD NATIONAL RESEARCH PARTNERSHIP | Improving practice through research

Maternal Health Phase 1

Feedback on priorities for improvement

Use a scale of 1-10, where 10 is most important and 1 is least important. The same ranking can be given to more than one priority – for example, if two priorities are equally 'most important', rate them both 10. If you think three are equally 'least important', rate all three as 1.

13. **Key information in client records/health summaries**

Improve recording of key client information and early pregnancy care across the scope of best practice, with a specific focus on the following:

	1 Least Important	2	3	4	5	6	7	8	9	10 Most Important
a. Folate supplementation prior to conception	○	○	○	○	○	○	○	○	○	○
b. Pregnancy care visits before 13 weeks gestation	○	○	○	○	○	○	○	○	○	○
c. Inclusion of a complete delivery summary sheet/letter in every medical record	○	○	○	○	○	○	○	○	○	○

14. **Risk factors and brief interventions**

Improve recording and delivery of risk factors and brief interventions across the scope of best practice, with a specific focus on:

	1 Least Important	2	3	4	5	6	7	8	9	10 Most Important
a. Enquiry about smoking and delivery of smoking cessation advice early in pregnancy	○	○	○	○	○	○	○	○	○	○
b. Enquiry about alcohol use and delivery of brief counselling early in pregnancy	○	○	○	○	○	○	○	○	○	○
c. Social risk factor assessment and, if evidence of social risk, record of referral to appropriate services	○	○	○	○	○	○	○	○	○	○
d. Discussion of benefits and appropriate preparation for and practice of breastfeeding	○	○	○	○	○	○	○	○	○	○
e. Discussion of domestic/social environment (including social/family support, domestic violence, substance abuse/misuse) and available support services	○	○	○	○	○	○	○	○	○	○
f. Discussion of financial situation, housing condition and food security, and available support services	○	○	○	○	○	○	○	○	○	○
g. Discussion of cultural considerations (social and cultural aspects of pregnancy and care: Women's Business)	○	○	○	○	○	○	○	○	○	○

15. **Laboratory investigations**

Improve recording and delivery of laboratory investigations across the scope of best practice, with a specific focus on:

	1 Least Important	2	3	4	5	6	7	8	9	10 Most Important
a. Discussing foetal anomaly screening available in the area with all women and their supports	○	○	○	○	○	○	○	○	○	○
b. Provision of anti-D injection if client is Rh factor negative with no antibodies at 28 and 34 weeks	○	○	○	○	○	○	○	○	○	○
c. Provision of Rubella immunisation post-birth if client has a low or negative Rubella titre during pregnancy	○	○	○	○	○	○	○	○	○	○

16. **Routine antenatal checks and abnormal findings**

Improve recording and delivery of routine antenatal checks and follow-up actions for abnormal findings across the scope of best practice, with a specific focus on:

	1 Least Important	2	3	4	5	6	7	8	9	10 Most Important
a. Calculating and recording Body Mass Index (BMI) in the first trimester	○	○	○	○	○	○	○	○	○	○
b. BMI management plans for all women	○	○	○	○	○	○	○	○	○	○
c. Mid-stream urine tests following abnormal urinalysis results	○	○	○	○	○	○	○	○	○	○
d. Performing cardiotocographs (CTG) in response to abnormal foetal movements	○	○	○	○	○	○	○	○	○	○
e. Initiating kick charts in response to abnormal foetal movements	○	○	○	○	○	○	○	○	○	○

17. **Emotional wellbeing screening and care**

Improve recording and delivery of emotional wellbeing screening and care across the scope of best practice, with a specific focus on:

	1 Least Important	2	3	4	5	6	7	8	9	10 Most Important
a. Emotional wellbeing screening for all women during pregnancy	○	○	○	○	○	○	○	○	○	○
b. Provision of appropriate follow-up for women identified as at-risk based on emotional wellbeing assessment	○	○	○	○	○	○	○	○	○	○

18. **Postnatal care:**
Improve recording and delivery of postnatal care across the scope of best practice, with a specific focus on:

	1 Least Important	2	3	4	5	6	7	8	9	10 Most Important
a. Discussion of smoking and the increased risk of SIDS in babies in a smoking environment	○	○	○	○	○	○	○	○	○	○
b. Discussion of SIDS prevention and the importance of keeping a safe environment for the baby	○	○	○	○	○	○	○	○	○	○
c. Discussion of diet and nutrition for the mother and baby	○	○	○	○	○	○	○	○	○	○
d. Discussion of infection prevention (washing hands, washing baby, nappy hygiene)	○	○	○	○	○	○	○	○	○	○
e. Discussion of mood, changes following birth and risk factors for postnatal depression	○	○	○	○	○	○	○	○	○	○
f. Discussion of domestic/social environment (including social/family support, domestic violence, substance abuse/misuse) and available support services	○	○	○	○	○	○	○	○	○	○
g. Discussion of financial situation, housing condition and food security, and available support services	○	○	○	○	○	○	○	○	○	○
h. Discussion of injury prevention and the importance of keeping a safe environment for the new mother	○	○	○	○	○	○	○	○	○	○

Feedback on priorities for improvement

19. **Health centre systems**

Improve system components that were scored relatively low by most health centres including:

	1 Least Important	2	3	4	5	6	7	8	9	10 Most Important
a. *'Links with the community'* to inform service and regional planning (in particular 'communication and cooperation on regional health planning,development of health resources, governance and operation of health centre'.	○	○	○	○	○	○	○	○	○	○
b. Specialist-generalist collaborations within the *'Information systems and decision support'* component	○	○	○	○	○	○	○	○	○	○
c. Continuity of care within the '*Delivery system design'* component	○	○	○	○	○	○	○	○	○	○
d. Physical infrastructure, supplies and equipment within the *'Delivery system design'* component	○	○	○	○	○	○	○	○	○	○
e. Education and support, behavioural risk reduction and peer support within the '*Self-Management Support'* component	○	○	○	○	○	○	○	○	○	○

20. For all the above items with the highest scores, please explain why you believe these should be the highest priorities for improvement (<u>excluding barriers and enablers, which will be addressed in Phase 2</u>).

21. A general priority in the report refers to focusing on health centres that are performing at relatively lower levels. Do you agree with this approach in general, or is it important to put effort into achieving improvement across all health centres regardless of performance? Please record your response on 1 to 5 scale, where 1 indicates a strong focus only on health centres with relatively low levels of performance and 5 indicates equal attention to all health centres regardless of performance.

- ○ 1 (strong focus on lower performing health centres)
- ○ 2
- ○ 3
- ○ 4
- ○ 5 (equal focus on all health centres regardless of performance)

NATIONAL RESEARCH PARTNERSHIP Improving practice through research

Maternal Health Phase 1

Feedback on General Priorities

22. To what extent are the priorities listed in the report consistent with your understanding of the priorities for improvement of maternal health care prior to reading this report?

- ○ Very consistent
- ○ Mostly consistent
- ○ Mostly not consistent
- ○ Not consistent at all

Please comment on how these priorities differ from what you previously regarded as priorities for improvement in maternal health care?

23. Has your view on priorities for improvement in maternal health care changed as a result of reading this report?

- ◯ Yes
- ◯ No

If yes, how your view has changed?

24. Are there any additional priorities that you/the group think should be listed in the report?

- ◯ Yes
- ◯ No

If yes, please list additional priorities and indicate why they are high priority

25. Should the priorities for your State/Territory be different to the priorities identified from the national maternal health care data?

- ◯ Yes
- ◯ No

If yes, please specify how and why the priorities for your State/Territory would differ from those arising from the national data?

ABCD NATIONAL RESEARCH PARTNERSHIP | Improving practice through research

Maternal Health Phase 1

FEEDBACK ON PHASE 1

26. Please give us your impression of the Phase 1 report: 'National Report on Aboriginal and Torres Strait Islander Maternal Health Care 2012 – 2014'

How well does the report:

	Very well	Well	Not well	Poorly
Present information in a way that is easy for you to read?	◯	◯	◯	◯
Present information in a way that is easy for you to use?	◯	◯	◯	◯
Provide information that is useful to you in your work?	◯	◯	◯	◯
Provide information that you would not otherwise have had access to?	◯	◯	◯	◯
Provide information that is credible?	◯	◯	◯	◯
Encourage discussion about improving specific aspects of maternal health care?	◯	◯	◯	◯
Encourage action to make improvements in specific aspects of maternal health care?	◯	◯	◯	◯

27. How could this survey have been improved?

28. A plain language summary was provided with the report. Do you have any feedback about the usefulness of this summary or suggestions for improvement?

29. A group facilitation guide was provided with the report, to support those leading group discussions about the ESP data. Do you have any feedback about the usefulness of this guide, or suggestions for improvement?

30. Other comments?

This is the end of the survey. Thank you for your time.

If you have any questions, please feel free to contact the ABCD National Research Partnership at abcd@menzies.edu.au
