# Supplementary material for: Identifying evidence-practice gaps and strategies for improvement in Aboriginal and Torres Strait Islander maternal health care
Source: PLoS One. 2018 Feb 7;13(2):e0192262. doi: 10.1371/journal.pone.0192262 (PMC5802899; doi:10.1371/journal.pone.0192262)
Supplement: S2 File — (PDF) [file pone.0192262.s002.pdf]

Maternal health - Phase 2 ESP Project

Introduction

Your participation in this survey will help us in identifying:

(1) barriers and enablers to addressing priority gaps in maternal health care

(2) strategies for improvement.

To enable this we ask you to reflect on the trend data presented in the Phase 2 ESP Project report, "*Priority Evidence-Practice Gaps in Aboriginal and Torres Strait Islander Maternal Health Care (with supporting data: 2007 – 2014) – Phase 2 ESP Project*", and the evidence brief on the barriers, enablers and strategies for using CQI to make improvements in primary health care quality. The evidence brief is in the report - Appendix F.

Where possible we have also developed separate reports of state/jurisdictional CQI data. These are available to people in those jurisdictions to allow comparison with the data in the national report. The work of the ABCD National Research Partnership is being conducted with the approval of research ethics committees in each of the jurisdictions where there are participating health centres. This survey will take about 25 minutes to complete.

* 1. By Clicking 'Yes' here, you consent to take part in this survey. Your responses will remain confidential.

- ◯ Yes, I agree to take part in this survey
- ◯ No, I don't want to take part in this survey

2. If you would like to receive feedback about this research, please provide your contact details:

Name	
Email address	
Phone	

If you wish to supply your details but not have them linked to your survey response, contact us separately at abcd@menzies.edu.au

Maternal health - Phase 2 ESP Project

Section 1 - Background Information

* 3. For which jurisdiction are you providing feedback? (You may select more than one option)

- [ ] National
- [ ] NSW
- [ ] SA
- [ ] QLD
- [ ] NSW
- [ ] WA
- [ ] NT
- [ ] Victoria
- [ ] Tasmania
- [ ] ACT

* 4. Which of the following best describes the population that you/the group are thinking about in relation to this survey? (You may select more than one option.)

- [ ] Urban
- [ ] Regional
- [ ] Remote

* 5. Are you responding on your own behalf, or on behalf of a group?

- ○ Individual
- ○ Group

ABCD NATIONAL RESEARCH PARTNERSHIP | Improving practice through research

Maternal health - Phase 2 ESP Project

Section 1 - Group Feedback

* 6. What proportion of the group identify as Aboriginal and/or Torres Strait Islander?

- ◯ All
- ◯ A Majority (more than half)
- ◯ A minority (less than half)
- ◯ None

7. How many people (approximately) were in the group?

* 8. What types of organisation did the members of the group come from? (You may select as many as relevant)

- ☐ Community controlled health centre
- ☐ Community controlled peak body
- ☐ Government health centre
- ☐ Government health department
- ☐ Primary Health Care Network
- ☐ General Practice
- ☐ University or Research organisation
- ☐ Other (please specify)

* 9. Which of the following roles were represented in the group? (You may select as many as relevant)

- [ ] Nurse
- [ ] Midwife
- [ ] Middle manager
- [ ] Doctor
- [ ] Public health physician
- [ ] Other medical specialist
- [ ] Senior management/executive
- [ ] CQI facilitator
- [ ] Board Member
- [ ] Policy Officer
- [ ] Aboriginal and/or Torres Islander health practitioner/worker
- [ ] Research/Academic
- [ ] Other (please specify)

Section 1 - Individual Feedback

* 10. If you are completing this survey as an individual, please indicate in which part of the health sector you are involved:

- Community controlled health centre
- Community controlled peak body
- Government health centre
- Government health department
- Primary Health Care Network
- General Practice
- University or Research organisation
- Other (please specify)

* 11. Which of the following best describes your role?

- Nurse
- Midwife
- Middle manager
- Doctor
- Public health physician
- Other medical specialist
- Senior management/executive
- CQI facilitator
- Board member
- Policy officer
- Aboriginal and/or Torres Strait Islander health practitioner/worker
- Researcher/ Academic
- Other (please specify)

* 12. Do you identify as being of Aboriginal and/or Torres Strait Islander origin?

- Yes
- No

Priority evidence-practice gaps

The priority evidence-practice gaps in maternal care identified through Phase 1 of the ESP Project, and detailed in the trend data report are:

# Enquiry about smoking and delivery of smoking cessation advice in pregnancy
# Enquiry about alcohol use and delivery of brief counselling early in pregnancy
# Social risk factor assessment in pregnancy and, if evidence of social risk, record of referral to appropriate services
# Provision of appropriate follow-up for women identified as at-risk based on emotional wellbeing assessment
# Discussion of SIDS prevention and the importance of keeping a safe environment for the baby (postnatal care)
# Discussion of diet and nutrition for the mother and baby (postnatal care)
# Emotional wellbeing screening for all women during pregnancy
# Discussion of smoking and the increased risk of SIDS in babies in a smoking environment (postnatal care)

We would like you to reflect on these priority evidence-practice gaps and the trend data when responding to the next section of the survey.

NATIONAL RESEARCH PARTNERSHIP | Improving practice through research
Maternal health - Phase 2 ESP Project

Section 2: Barriers and Enablers to Addressing Gaps

The next sections cover aspects of health centre systems, the broader system environment and staff attributes identified in international and Australian research as having the potential to impact on quality of care in a variety of contexts. The survey seeks your opinion on the extent to which the various items identified may be important in the Aboriginal and Torres Strait Islander primary health care (PHC) context.

Your responses should relate to:
(1) providing best practice maternal health care as relevant to the priority evidence-practice gaps.
(2) reflections on the report *'Priority Evidence-Practice Gaps in Aboriginal and Torres Strait Islander Maternal Health Care (with supporting data: 2007 – 2014) - Phase 2 ESP Project'*
(3) the PHC system for Aboriginal and Torres Strait Islander people in general rather than to any

specific health centre or service.

Attributes of the health centre or broader health system environment that support best practice in maternal health care.

Please select responses to the following statements.

Financing and resources

13. Within the socio-political context, there is sufficient financial support (e.g. from local/regional health authorities, government) to support best practice in maternal health care as relevant to the priority evidence-practice gaps

Strongly disagree	Partly disagree	Partly agree	Strongly agree	Don't know
○	○	○	○	○

Facilities and equipment

14. PHC facilities are generally of adequate size, design, and condition

Strongly disagree	Partly disagree	Partly agree	Strongly agree	Don't know
○	○	○	○	○

15. PHC facilities generally have adequate equipment

Strongly disagree	Partly disagree	Partly agree	Strongly agree	Don't know
○	○	○	○	○

Staffing / workforce

16. PHC centres generally have adequate numbers of staff

Strongly disagree	Partly disagree	Partly agree	Strongly agree	Don't know
○	○	○	○	○

17. PHC centres generally have appropriate types of each of the following categories of staff

	Strongly disagree	Partly disagree	Partly agree	Strongly agree	Don't know
Nurses	○	○	○	○	○
Aboriginal or Torres Strait Islander Health Practitioners/Workers	○	○	○	○	○
Doctors (GPs)	○	○	○	○	○
Medical Specialists	○	○	○	○	○
Allied health workers	○	○	○	○	○
Administrative staff	○	○	○	○	○

Staff/ workforce support, recruitment, and retention

18. There are good systems in place to ensure PHC staff have support from experienced staff, especially when health centres are affected by turnover of staff and staff shortages

Strongly disagree	Partly disagree	Partly agree	Strongly agree	Don't know
○	○	○	○	○

19. There are good systems in place to recruit, retain and support each of the following categories of staff (full time or visiting) as integral members of PHC teams

	Strongly disagree	Partly disagree	Partly agree	Strongly agree	Don't know
Nurses	○	○	○	○	○
Aboriginal or Torres Strait Islander Health Practitioners/Workers	○	○	○	○	○
Doctors (GPs)	○	○	○	○	○
Medical Specialists	○	○	○	○	○
Allied health workers	○	○	○	○	○
Administrative staff	○	○	○	○	○

Teamwork

20. PHC staff function effectively in teams

Strongly disagree	Partly disagree	Partly agree	Strongly agree	Don't know
○	○	○	○	○

21. PHC staff are generally clear about their roles in relation to other members of the PHC team

Strongly disagree	Partly disagree	Partly agree	Strongly agree	Don't know
◯	◯	◯	◯	◯

22. PHC staff are trained to work effectively in teams

Strongly disagree	Partly disagree	Partly agree	Strongly agree	Don't know
◯	◯	◯	◯	◯

In-service training and development

23. There are good systems in place to support staff development, including the development of knowledge and skills required

Strongly disagree	Partly disagree	Partly agree	Strongly agree	Don't know
◯	◯	◯	◯	◯

24. There are good systems in place to support inter-organisational and intra-organisational learning

Strongly disagree	Partly disagree	Partly agree	Strongly agree	Don't know
◯	◯	◯	◯	◯

Self-management

25. Staff are well trained in the principles of client self-management as relevant to maternal health care

Strongly disagree	Partly disagree	Partly agree	Strongly agree	Don't know
◯	◯	◯	◯	◯

26. There are good self-management resources that are relevant to maternal health care available to PHC staff

Strongly disagree	Partly disagree	Partly agree	Strongly agree	Don't know
◯	◯	◯	◯	◯

ABCD NATIONAL RESEARCH PARTNERSHIP | Improving practice through research

Maternal health - Phase 2 ESP Project

Section 2 continued - Barriers and Enablers to Addressing Gaps

Patient-centered care

27. There are good systems in place to support all members of PHC teams in understanding the needs and aspirations of people living in Aboriginal and Torres Strait Islander communities for the purpose of providing best practice maternal health care

Strongly disagree	Partly disagree	Partly agree	Strongly agree	Don't know
○	○	○	○	○

28. There are good systems in place to support all members of PHC teams to provide care that is respectful of and responsive to individual patient preferences, needs, and values, and ensuring that patient values guide all clinical decisions

Strongly disagree	Partly disagree	Partly agree	Strongly agree	Don't know
○	○	○	○	○

29. There are good systems in place to train all members of PHC teams in providing patient-centered care for people living in Aboriginal and Torres Strait Islander communities

Strongly disagree	Partly disagree	Partly agree	Strongly agree	Don't know
○	○	○	○	○

Population health

30. There are good systems in place to ensure PHC teams have a clear understanding of the size, diversity and other key features of their service populations and to apply this knowledge

Strongly disagree	Partly disagree	Partly agree	Strongly agree	Don't know
○	○	○	○	○

31. There are good systems in place to ensure PHC teams are able to apply the principles of population health

Strongly disagree	Partly disagree	Partly agree	Strongly agree	Don't know
○	○	○	○	○

32. There are good systems in place to ensure PHC teams are well trained in the principles of population health

Strongly disagree	Partly disagree	Partly agree	Strongly agree	Don't know
○	○	○	○	○

Decision support

33. Best practice guidelines and other decision support resources are available to PHC staff

Strongly disagree	Partly disagree	Partly agree	Strongly agree	Don't know
○	○	○	○	○

34. PHC staff are adequately trained to use the available best practice guidelines and other decision support resources

Strongly disagree	Partly disagree	Partly agree	Strongly agree	Don't know
○	○	○	○	○

Clinical information systems and communication technology

35. The clinical information systems and communication technology in place have the functionality to support provision of best practice care

Strongly disagree	Partly disagree	Partly agree	Strongly agree	Don't know
○	○	○	○	○

36. PHC staff are trained and effectively supported to use clinical information systems and communication technology for supporting and providing best practice

Strongly disagree	Partly disagree	Partly agree	Strongly agree	Don't know
○	○	○	○	○

Quality Improvement

37. There are good quality improvement tools available in health centres for supporting and improving delivery of best practice care

Strongly disagree	Partly disagree	Partly agree	Strongly agree	Don't know
○	○	○	○	○

38. There are good processes in place to support health centre staff to interpret quality improvement data, plan and implement strategies for improvement

Strongly disagree	Partly disagree	Partly agree	Strongly agree	Don't know
○	○	○	○	○

39. PHC staff are adequately trained to use quality improvement tools and resources for supporting and improving delivery of best practice care

Strongly disagree	Partly disagree	Partly agree	Strongly agree	Don't know
○	○	○	○	○

40. Managers are adequately trained to support effective use of quality improvement tools and resources for monitoring and enhancing delivery of best practice care

Strongly disagree	Partly disagree	Partly agree	Strongly agree	Don't know
○	○	○	○	○

41. There is good local ownership by PHC staff of CQI data and CQI processes for supporting and improving delivery of best practice care

Strongly disagree	Partly disagree	Partly agree	Strongly agree	Don't know
○	○	○	○	○

42. Managers provide clear and appropriate support for effective use of quality improvement tools and resources by PHC staff for monitoring and enhancing delivery of best practice care

Strongly disagree	Partly disagree	Partly agree	Strongly agree	Don't know
○	○	○	○	○

43. PHC staff generally believe that CQI data and CQI processes can be used for supporting and improving delivery of best practice

Strongly disagree	Partly disagree	Partly agree	Strongly agree	Don't know
○	○	○	○	○

Community capacity, engagement, mobilisation

44. There are good systems in place to increase the expectation of community members with regard to best practice care

Strongly disagree	Partly disagree	Partly agree	Strongly agree	Don't know
○	○	○	○	○

45. There are good systems in place to strengthen community leadership for quality with regard to best practice.

Strongly disagree	Partly disagree	Partly agree	Strongly agree	Don't know
○	○	○	○	○

46. There are good systems in place to enhance the health literacy of community members with regard to best practice care

Strongly disagree	Partly disagree	Partly agree	Strongly agree	Don't know
○	○	○	○	○

47. There are good systems in place to build the capability and to support PHC staff to develop effective links to work in partnership with the communities they serve in providing best practice care.

Strongly disagree	Partly disagree	Partly agree	Strongly agree	Don't know
○	○	○	○	○

Leadership and management

48. There is good clinical and management leadership at the regional, state, national level for supporting and providing best practice care

Strongly disagree	Partly disagree	Partly agree	Strongly agree	Don't know
◯	◯	◯	◯	◯

49. There are good networks and regional coordination between parties involved in supporting and providing best practice care

Strongly disagree	Partly disagree	Partly agree	Strongly agree	Don't know
◯	◯	◯	◯	◯

50. Managers actively support the development of partnerships across the health sector for the purpose of enhancing delivery of best practice care

Strongly disagree	Partly disagree	Partly agree	Strongly agree	Don't know
◯	◯	◯	◯	◯

51. Please provide additional comments here on aspects of the**health system or health centre environment** that are significant enablers or barriers to providing best practice maternal health care as relevant to any or all of the priority evidence-practice gaps

Section 2 continued: Barriers and Enablers to Addressing Gaps

Attributes of health centre staff that support best practice in maternal health care

Knowledge

52. PHC staff know the content and objectives of best practice care for maternal health

Strongly disagree	Partly disagree	Partly agree	Strongly agree	Don't know
◯	◯	◯	◯	◯

53. PHC staff are aware of how to provide best practice maternal health care for Aboriginal and Torres Strait Islander women

Strongly disagree	Partly disagree	Partly agree	Strongly agree	Don't know
○	○	○	○	○

Skills

54. PHC staff have the skills to provide best practice maternal health care for Aboriginal and Torres Strait Islander women

Strongly disagree	Partly disagree	Partly agree	Strongly agree	Don't know
○	○	○	○	○

Social/ professional role and identity

55. PHC staff recognise that it is their professional responsibility to provide best practice maternal health care for Aboriginal and Torres Strait Islander women

Strongly disagree	Partly disagree	Partly agree	Strongly agree	Don't know
○	○	○	○	○

Beliefs about capabilities

56. PHC staff are confident in their ability to provide best practice maternal health care for Aboriginal and Torres Strait Islander women

Strongly disagree	Partly disagree	Partly agree	Strongly agree	Don't know
○	○	○	○	○

Optimism

57. With regard to providing best practice maternal health care for Aboriginal and Torres Strait Islander women, PHC staff are optimistic about the future

Strongly disagree	Partly disagree	Partly agree	Strongly agree	Don't know
○	○	○	○	○

Beliefs about consequences

58. PHC staff believe that if they provide best practice care for Aboriginal and Torres Strait Islander women who are pregnant, it will have benefits for the health of Aboriginal and Torres Strait Islander people more generally at a population level

Strongly disagree	Partly disagree	Partly agree	Strongly agree	Don't know
○	○	○	○	○

59. PHC staff believe that if they provide best practice care for Aboriginal and Torres Strait Islander women it will have disadvantages for their relationships with Aboriginal and Torres Strait Islander people.

Strongly disagree	Partly disagree	Partly agree	Strongly agree	Don't know
○	○	○	○	○

Intentions

60. For every 10 Aboriginal and Torres Strait Islander women attending PHC services for maternal health care, for how many would the PHC staff (on average) intend to provide best practice maternal health care?

- ○ 1
- ○ 2
- ○ 3
- ○ 4
- ○ 5
- ○ 6
- ○ 7
- ○ 8
- ○ 9
- ○ 10

61. How strong is the intention of PHC staff to provide best practice care every day to Aboriginal and Torres Strait Islander women attending their services?

Not strong at all	Quite stong	Mostly Strong	Always very strong	Don't know
○	○	○	○	○

Memory, attention and decision processes

62. How often do PHC staff remember to provide best practice maternal health care for Aboriginal and Torres Strait Islander women who are pregnant?

Never	Occasionally	Often	Always	Don't know
○	○	○	○	○

63. PHC staff believe that when they need to concentrate to provide best practice maternal health care for Aboriginal and Torres Strait Islander women, they have no trouble focusing their attention

Strongly disagree	Partly disagree	Partly agree	Strongly agree	Don't know
○	○	○	○	○

Social Influences

64. Most people of influence in Aboriginal and Torres Strait Islander PHC services are seen by PHC staff to support the provision of best practice care for Aboriginal and Torres Strait Islander women

Strongly disagree	Partly disagree	Partly agree	Strongly agree	Don't know
○	○	○	○	○

Emotion

65. Are PHC staff who provide maternal health care for Aboriginal and Torres Strait Islander women currently able to enjoy their normal day-to-day work activities?

Never	Occasionally	Often	Always	Don't know
○	○	○	○	○

66. Do PHC staff who currently provide maternal health care for Aboriginal and Torres Strait Islander women feel unhappy, anxious or depressed about their work?

Never	Occasionally	Often	Always	Don't know
○	○	○	○	○

67. Please provide additional comments here on **staff attributes that are significant enablers or barriers** to providing best practice maternal health care as relevant to any or all of the priority evidence-practice gaps.

Section 2 continued: Barriers and Enablers to Addressing Gaps

68. Please rate the relative importance of the **health centre and health system attributes** and the **staff attributes** to achieving improvements in the areas of priority evidence-practice gaps. Indicate a score between 1 and 7.

1. **Health centre and health system attributes** are of over-riding importance			4. Equal importance			7. **Staff attributes** are of over-riding importance
○	○	○	○	○	○	○

NATIONAL RESEARCH PARTNERSHIP | Improving practice through research

Maternal health - Phase 2 ESP Project

Section 3 - Strategies for Improvement

Please suggest strategies and actions that could be used to address barriers and enablers for each priority evidence-practice gap below:

69. Enquiry about smoking and delivery of smoking cessation advice in pregnancy

70. Enquiry about alcohol use and delivery of brief counselling early in pregnancy

71. Social risk factor assessment in pregnancy and, if evidence of social risk, record of referral to appropriate services

72. Provision of appropriate follow-up for women identified as at-risk based on emotional wellbeing assessment

73. Discussion of SIDS prevention and the importance of keeping a safe environment for the baby (postnatal care)

74. Discussion of diet and nutrition for the mother and baby (postnatal care)

75. Emotional wellbeing screening for all women during pregnancy

76. Discussion of smoking and the increased risk of SIDS in babies in a smoking environment (postnatal care)

77. Overall maternal care

Maternal health - Phase 2 ESP Project

Section 3 continued: Strategies for improvement

Considering your responses to questions about strategies and actions to address the evidence-practice gaps:

78. What could you do in your current professional role to support the development and implementation of these strategies and actions?

79. What support can you give others in your organisation, and in other organisations, to help them implement these strategies and actions?

80. What measures and processes could be used to monitor change over time in the priority evidence-practice gaps, in order to understand how well the strategies are working?

81. What support do you need from colleagues and managers in your organisation, and in other organisations, so you can help implement these strategies and actions?

Maternal health - Phase 2 ESP Project

Section 4 - Feedback on how information was presented

We are seeking feedback on how information was presented in the report on*'Priority Evidence-Practice Gaps in Aboriginal and Torres Strait Islander Maternal Health Care (with supporting data: 2007 – 2014) - Phase 2 ESP Project'*. Your feedback will help us improve future reports.

82. Overall, how well does the report:

	Poorly	Not very well	Well	Very Well
Present information in a way that is easy for you to read and understand?	○	○	○	○
Present information in a way that is easy for you to use?	○	○	○	○
Provide information that is useful to you?	○	○	○	○
Encourage discussion about the barriers and enablers for addressing the priority evidence-practice gaps for maternal health care?	○	○	○	○

83. Do you have suggestions about how to improve the presentation and usefulness of the report, or how it could be changed to encourage discussion?

84. Do you have any feedback about how to improve the evidence brief provided with the report? [Appendix G, Improving the Quality of Aboriginal and Torres Strait Islander primary health care – What the research shows]

85. A group facilitation guide was provided with the report, to support those leading group discussions about the ESP data. Do you have any feedback about the usefulness of this guide, or suggestions for improvement?

86. A plain language summary was provided with the report. Do you have any feedback about the usefulness of this summary or suggestions for improvement?

Section 5: Feedback on this survey

We have asked you to identify barrier, enablers and strategies in one survey, and will feed this information back in one report. (This occurred over two phases of reports and feedback in the earlier ESP Project cycles for child health and chronic illness care).

87. Do you have any comments about this changed approach?

88. Do you have any comments or suggestions about how to improve future ESP Project surveys?

THANK YOU

This is the end of the survey. Thank you for your time. If you have any questions, please feel free to contact the ABCD National Research Partnership at ABCD
