# Supplementary material for: Identifying evidence-practice gaps and strategies for improvement in Aboriginal and Torres Strait Islander maternal health care
Source: PLoS One. 2018 Feb 7;13(2):e0192262. doi: 10.1371/journal.pone.0192262 (PMC5802899; doi:10.1371/journal.pone.0192262)
Supplement: S3 File — (PDF) [file pone.0192262.s003.pdf]

Maternal Health Draft Final Report

Introduction

This is the final survey in the phased ESP Project about maternal health care.

Its purpose is to:

a) confirm that we have accurately reflected the feedback about the barriers and enablers to addressing the identified gaps in care and strategies for improvement
b) gather any additional comments on the ESP Project processes for consideration in dissemination of findings.

To complete this survey, you need access to the 'Priority Evidence-Practice Gaps in Aboriginal and Torres Strait Islander Maternal Health Care - Draft Final Report'

The ABCD National Research Partnership is conducted with the approval of research ethics committees in each of the jurisdictions where there are participating health centres.

An asterix '*' next to the question number indicates that a response is required. This survey will take about 10 minutes to complete.

* 1. By clicking 'Yes' here, you consent to take part in this survey. Your responses will remain confidential.

- ◯ Yes, I agree to take part in this survey
- ◯ No, I don't want to take part in this survey

Maternal Health Draft Final Report

Background Information

Please provide some information about yourself.

* 2. Which jurisdiction are you considering for this feedback? (select all that apply)

- [ ] Australia
- [ ] ACT
- [ ] New South Wales
- [ ] Northern Territory
- [ ] Queensland
- [ ] South Australia
- [ ] Tasmania
- [ ] Western Australia
- [ ] Victoria

* 3. What is the location of the population group you are considering in your feedback? (select all that apply)

- [ ] Urban
- [ ] Regional
- [ ] Remote

* 4. Are you responding on your own behalf, or on behalf of a group?

- ○ On my own behalf
- ○ Group (two or more people who met, talked about the report and agreed on the responses)

Group Background

* 5. If you are responding on behalf of a group:

How many people met and talked about the report and agreed on the responses given here?

* 6. What proportion of the group identify as Aboriginal and/or Torres Strait Islander?

- ◯ All
- ◯ More than half
- ◯ Less than half
- ◯ None

* 7. Select roles represented by group members (select all that apply)

- ☐ Nurse
- ☐ Middle manager
- ☐ Midwife
- ☐ Medical Officer/General Practitioner/Registrar
- ☐ Obstetrician/gynaecologist
- ☐ Other medical specialist
- ☐ Senior manager/Executive
- ☐ CQI facilitator
- ☐ Board member
- ☐ Policy officer
- ☐ Aboriginal and/or Torres Strait Islander health practitioner/worker
- ☐ Researcher/Academic
- ☐ Other

Other (please specify)

* 8. What types of organisations do the members of the group represent? (select all that apply)

- [ ] Community controlled health centre
- [ ] Community controlled peak body
- [ ] Government health centre
- [ ] Government health department
- [ ] Primary Health Care Network
- [ ] General Practice
- [ ] University or Research Organisation
- [ ] Other

Other (please specify)

Individual Background

* 9. If you are responding as an individual:

Do you identify as being of Aboriginal and/or Torres Strait Islander origin?

- ○ Yes
- ○ No

* 10. What is your primary role?

- Nurse
- Midwife
- Middle manager
- Medical Officer/General Practitioner/Registrar
- Obstetrician/gynaecologist
- Other medical specialist
- Senior manager/Executive
- CQI facilitator
- Board member
- Policy officer
- Aboriginal and/or Torres Strait Islander health practitioner/worker
- Researcher/Academic
- Other

Other (please specify)

* 11. What types of organisations do you represent?

- Community controlled health centre
- Community controlled peak body
- Government health centre
- Government health department
- Primary Health Care Network
- General Practice
- University or Research Organisation
- Other

Other (please specify)

Feedback on barriers, enablers and strategies

12. Do you have any additional comments and/or suggestions on the **barriers and enablers** to addressing the identified gaps in preventive care?

13. Do you have any additional comments and/or suggestions for**strategies** to address the identified barriers and enablers to maternal care?

ABCD NATIONAL RESEARCH PARTNERSHIP | Improving practice through research

Maternal Health Draft Final Report

Feedback on presentation of information

14. We are interested in your views on presentation of information and content of this report. How well does the presentation of information and content of the report meet your needs?

- ◯ Very well
- ◯ Well
- ◯ Not very well
- ◯ Poorly

If your response to the above question was ‘Poorly’ or ‘Not very well’, then please provide suggestions for how the presentation could be improved.

ABCD NATIONAL RESEARCH PARTNERSHIP | Improving practice through research

Maternal Health Draft Final Report

Feedback on the ESP Project processes

We have used a phased approach to encourage engagement with data by key stakeholders, and to identify barriers, enablers and strategies to address priority evidence practice gaps. Your feedback on the ESP processes for maternal care will help us to refine ESP processes for other areas of care.

15. Has the cyclical process of engagement improved your understanding of using aggregated data to inform decision making? Please provide comment.

16. Have you used the aggregated CQI data provided through this process? If so, how?

17. Have you used the knowledge and ideas shared by other stakeholders through this process? If so, how?

18. To help us improve the ESP process, please feed back any stakeholder perceptions of the ESP project that may not have been captured in survey responses (e.g. in workplace discussions).

19. Please provide suggestions for dissemination of the Final Maternal Care report.

20. We would welcome any other suggestions for improving the ESP project approach or processes.

Thankyou

21. The information gathered through this survey will be reported in a way that does not identify individuals or specific groups. However, we would like to send you a copy of the Final Maternal Care Report. We may also wish to follow up with additional questions to clarify responses.
If you are happy to provide your contact details for these purposes, please enter them below.

If you do not want your contact details linked to your responses, but would still like to receive feedback and remain involved, you can email your contact details to abcd@menzies.edu.au

This is the end of the survey. Thank you for your time.

If you have any questions, please feel free to contact the ABCD National Research Partnership at abcd@menzies.edu.au

Name: ____________________

Email: ____________________
