# Supplementary material for: Identifying evidence-practice gaps and strategies for improvement in Aboriginal and Torres Strait Islander maternal health care
Source: PLoS One. 2018 Feb 7;13(2):e0192262. doi: 10.1371/journal.pone.0192262 (PMC5802899; doi:10.1371/journal.pone.0192262)
Supplement: S1 Table — PHC: primary health care, CQI: continuous quality improvement, n: number. (DOCX) [file pone.0192262.s004.docx]

**S1 Table. Characteristics of PHC centres providing maternal health audit data from 2007 to 2014.** PHC: primary health care, CQI: continuous quality improvement, n: number

| **Year** | **2007** | **2008** | **2009** | **2010** | **2011** | **2012** | **2013** | **2014** | **Total** |
| --- | --- | --- | --- | --- | --- | --- | --- | --- | --- |
| **Number of PHC centres** | 19 | 24 | 29 | 28 | 43 | 50 | 36 | 14 | 91 |
| **Location: n (%)** |  | | | | | | | | |
| Urban/Regional | 5 (26) | 8 (33) | 8 (27) | 5 (18) | 11 (26) | 7 (14) | 9 (25) | 4 (29) | 22 (24) |
| Remote | 14 (74) | 16 (67) | 21 (72) | 23 (82) | 32 (74) | 43 (86) | 27 (75) | 10 (71) | 69 (76) |
| **Governance: n (%)** |  | | | | | | | | |
| Government | 5 (26) | 9 (38) | 16 (55) | 20 (71) | 32 (74) | 38 (76) | 27 (75) | 9 (64) | 63 (69) |
| Community | 14 (74) | 15 (63) | 13 (45) | 8 (29) | 11 (26) | 12 (24) | 9 (25) | 5 (36) | 28 (31) |
| **Population size: n (%)** |  | | | | | | | | |
| <1000 | 10 (53) | 10 (42) | 11 (38) | 12 (43) | 22 (51) | 28 (56) | 14 (39) | 3 (21) | 44 (48) |
| ≥1000 | 9 (47) | 14 (58) | 18 (62) | 16 (57) | 21 (49) | 22 (44) | 22 (61) | 11 (79) | 47 (52) |
| **CQI cycles: n (%)** |  | | | | | | | | |
| Baseline only (1 audit) | 19 (100) | 11 (46) | 11 (38) | 10 (36) | 21 (49) | 12 (23) | 5 (14) | 2 (14) | 18 (31) |
| 1 cycle (2 audits) |  | 13 (54) | 10 (34) | 10 (36) | 8 (19) | 14 (27) | 6 (17) | 2 (14) | 18 (20) |
| ≥2 cycles |  |  | 8 (28) | 8 (29) | 14 (33) | 26 (50) | 25 (69) | 10 (71) | 45 (49) |
| **Number of audited records** | 342 | 387 | 498 | 442 | 821 | 892 | 669 | 351 | 4402 |
